# Supplementary material for: Analysis of the impact of expressway construction on soil moisture in road areas
Source: PLoS One. 2023 Mar 30;18(3):e0283225. doi: 10.1371/journal.pone.0283225 (PMC10062659; doi:10.1371/journal.pone.0283225)
Supplement: S1 Table — (DOCX) [file pone.0283225.s001.docx]

**S1 Table. Annual average VSWI within the 1km radius of each interchange node before and after its construction**

| **state**  **node** | | **Last year** | **During construction** | **Finished** |
| --- | --- | --- | --- | --- |
| 1 | Year | 2005 | 2006 | 2007 |
|  | Value | 0.02082 | 0.02016 | 0.01987 |
| 2 | Year | 2005 | 2006 | 2007 |
|  | Value | 0.01633 | 0.01577 | 0.01547 |
| 3 | Year | 2008 | 2009 | 2010 |
|  | Value | 0.01263 | 0.01203 | 0.00964 |
| 4 | Year | 2005 | 2006 | 2007 |
|  | Value | 0.01721 | 0.01611 | 0.01594 |
| 5 | Year | 2005 | 2006 | 2007 |
|  | Value | 0.01297 | 0.01112 | 0.01134 |
| 6 | Year | 2005 | 2006 | 2007 |
|  | Value | 0.00933 | 0.00799 | 0.00763 |
| 7 | Year | 2005 | 2006 | 2007 |
|  | Value | 0.01746 | 0.01513 | 0.01488 |
| 8 | Year | 2005 | 2006 | 2007 |
|  | Value | 0.01634 | 0.01228 | 0.01256 |
| 9 | Year | 2014 | 2015 | 2016 |
|  | Value | 0.01903 | 0.01063 | 0.01572 |
| 10 | Year | 2008 | 2009 | 2010 |
|  | Value | 0.01168 | 0.01117 | 0.00683 |
| 11 | Year | 2008 | 2009 | 2010 |
|  | Value | 0.01579 | 0.01518 | 0.00615 |
| 12 | Year | 2014 | 2015 | 2016 |
|  | Value | 0.01339 | 0.01272 | 0.01452 |
