# Supplementary material for: Analysis of the impact of expressway construction on soil moisture in road areas
Source: PLoS One. 2023 Mar 30;18(3):e0283225. doi: 10.1371/journal.pone.0283225 (PMC10062659; doi:10.1371/journal.pone.0283225)
Supplement: S2 Table — (DOCX) [file pone.0283225.s002.docx]

**S2 Table. Annual average VSWI within the radius of each interchange node of Yongjin Expressway before and after its construction**

| **Point** | **Buffer distance/m** | **2005** | **2006** | **2007** |
| --- | --- | --- | --- | --- |
| 1 | 0-200 | 0.01664 | 0.01721 | 0.01682 |
|  | 200-500 | 0.02012 | 0.01956 | 0.01777 |
|  | 500-1000 | 0.02082 | 0.02016 | 0.01987 |
|  | 1000-2000 | 0.02122 | 0.02292 | 0.02036 |
|  | 2000-5000 | 0.01971 | 0.02312 | 0.02281 |
| 2 | 0-200 | 0.01413 | 0.01384 | 0.01402 |
|  | 200-500 | 0.01491 | 0.01422 | 0.01458 |
|  | 500-1000 | 0.01633 | 0.01577 | 0.01547 |
|  | 1000-2000 | 0.01561 | 0.01618 | 0.01648 |
|  | 2000-5000 | 0.01873 | 0.01711 | 0.01699 |
| 4 | 0-200 | 0.01223 | 0.01571 | 0.01568 |
|  | 200-500 | 0.01681 | 0.01589 | 0.01583 |
|  | 500-1000 | 0.01721 | 0.01611 | 0.01594 |
|  | 1000-2000 | 0.01788 | 0.01688 | 0.01695 |
|  | 2000-5000 | 0.01885 | 0.01728 | 0.01744 |
| 5 | 0-200 | 0.0107 | 0.01011 | 0.01113 |
|  | 200-500 | 0.01212 | 0.01102 | 0.01135 |
|  | 500-1000 | 0.01297 | 0.01112 | 0.01134 |
|  | 1000-2000 | 0.01414 | 0.01233 | 0.01285 |
|  | 2000-5000 | 0.01451 | 0.01316 | 0.01367 |
| 6 | 0-200 | 0.00485 | 0.00621 | 0.00656 |
|  | 200-500 | 0.00688 | 0.00711 | 0.00711 |
|  | 500-1000 | 0.00933 | 0.00799 | 0.00763 |
|  | 1000-2000 | 0.0106 | 0.0112 | 0.01321 |
|  | 2000-5000 | 0.01352 | 0.01452 | 0.01426 |
| 7 | 0-200 | 0.02302 | 0.01445 | 0.01411 |
|  | 200-500 | 0.02093 | 0.01488 | 0.01453 |
|  | 500-1000 | 0.01746 | 0.01513 | 0.01488 |
|  | 1000-2000 | 0.01722 | 0.01612 | 0.01523 |
|  | 2000-5000 | 0.01643 | 0.01754 | 0.01611 |
| 8 | 0-200 | 0.02085 | 0.01211 | 0.01188 |
|  | 200-500 | 0.01877 | 0.01221 | 0.01226 |
|  | 500-1000 | 0.01634 | 0.01228 | 0.01256 |
|  | 1000-2000 | 0.01622 | 0.01299 | 0.01319 |
|  | 2000-5000 | 0.01511 | 0.01379 | 0.01366 |
