# Supplementary material for: Analysis of the impact of expressway construction on soil moisture in road areas
Source: PLoS One. 2023 Mar 30;18(3):e0283225. doi: 10.1371/journal.pone.0283225 (PMC10062659; doi:10.1371/journal.pone.0283225)
Supplement: S3 Table — (DOCX) [file pone.0283225.s003.docx]

**S3 Table. Annual average VSWI within the radius of each interchange node before and after the construction of the Jinliwin Expressway**

| **Point** | **Buffer distance/m** | **2005** | **2006** | **2007** |
| --- | --- | --- | --- | --- |
| 3 | 0-200 | 0.01249 | 0.01432 | 0.00866 |
|  | 200-500 | 0.01287 | 0.01263 | 0.00879 |
|  | 500-1000 | 0.01263 | 0.01203 | 0.00964 |
|  | 1000-2000 | 0.01283 | 0.01246 | 0.00976 |
|  | 2000-5000 | 0.01387 | 0.01493 | 0.01244 |
| 10 | 0-200 | 0.0106 | 0.01031 | 0.00543 |
|  | 200-500 | 0.01042 | 0.00957 | 0.00471 |
|  | 500-1000 | 0.01168 | 0.01117 | 0.00683 |
|  | 1000-2000 | 0.01409 | 0.01333 | 0.01047 |
|  | 2000-5000 | 0.01495 | 0.01392 | 0.01466 |
| 11 | 0-200 | 0.01534 | 0.01494 | 0.00391 |
|  | 200-500 | 0.01448 | 0.01463 | 0.00486 |
|  | 500-1000 | 0.01579 | 0.01518 | 0.00615 |
|  | 1000-2000 | 0.01701 | 0.01652 | 0.00731 |
|  | 2000-5000 | 0.01744 | 0.01694 | 0.00845 |
