# Supplementary material for: Analysis of the impact of expressway construction on soil moisture in road areas
Source: PLoS One. 2023 Mar 30;18(3):e0283225. doi: 10.1371/journal.pone.0283225 (PMC10062659; doi:10.1371/journal.pone.0283225)
Supplement: S4 Table — (DOCX) [file pone.0283225.s004.docx]

**S4 Table. Annual average VSWI values in different radiation radii of each interchange node of Yongjin Expressway before and after its construction**

| **Point** | **Buffer distance/km** | **2005** | **2006** | **2007** | **2008** | **2009** |
| --- | --- | --- | --- | --- | --- | --- |
| 1 | 0-2 | 0.02122 | 0.02292 | 0.02036 | 0.01934 | 0.01837 |
|  | 2-4 | 0.01931 | 0.01843 | 0.01821 | 0.01730 | 0.01643 |
|  | 4-6 | 0.01969 | 0.01902 | 0.01846 | 0.01754 | 0.01666 |
|  | 6-8 | 0.02128 | 0.01960 | 0.01879 | 0.01785 | 0.01696 |
|  | 8-10 | 0.02015 | 0.20990 | 0.02101 | 0.02143 | 0.02186 |
| 2 | 0-2 | 0.01561 | 0.01618 | 0.01648 | 0.01599 | 0.01551 |
|  | 2-4 | 0.01876 | 0.01545 | 0.01522 | 0.01476 | 0.01432 |
|  | 4-6 | 0.01900 | 0.01701 | 0.01675 | 0.01625 | 0.01576 |
|  | 6-8 | 0.01934 | 0.01769 | 0.01699 | 0.01648 | 0.01599 |
|  | 8-10 | 0.01770 | 0.01809 | 0.01877 | 0.01915 | 0.01953 |
| 4 | 0-2 | 0.01788 | 0.01688 | 0.01695 | 0.01644 | 0.01595 |
|  | 2-4 | 0.01637 | 0.01349 | 0.01329 | 0.01289 | 0.01250 |
|  | 4-6 | 0.01658 | 0.01485 | 0.01462 | 0.01418 | 0.01376 |
|  | 6-8 | 0.01689 | 0.01545 | 0.01483 | 0.01439 | 0.01396 |
|  | 8-10 | 0.01545 | 0.01579 | 0.01639 | 0.01671 | 0.01705 |
| 5 | 0-2 | 0.01414 | 0.01233 | 0.01285 | 0.01246 | 0.01209 |
|  | 2-4 | 0.01113 | 0.00917 | 0.00904 | 0.00876 | 0.00850 |
|  | 4-6 | 0.01128 | 0.01010 | 0.00994 | 0.00965 | 0.00936 |
|  | 6-8 | 0.01148 | 0.01050 | 0.01009 | 0.00978 | 0.00949 |
|  | 8-10 | 0.01051 | 0.01074 | 0.01114 | 0.01137 | 0.01159 |
| 6 | 0-2 | 0.01060 | 0.01043 | 0.00988 | 0.00958 | 0.00930 |
|  | 2-4 | 0.01015 | 0.00836 | 0.00824 | 0.00799 | 0.00775 |
|  | 4-6 | 0.01028 | 0.00921 | 0.00907 | 0.00879 | 0.00853 |
|  | 6-8 | 0.01047 | 0.00958 | 0.00920 | 0.00892 | 0.00865 |
|  | 8-10 | 0.00958 | 0.00979 | 0.01016 | 0.01036 | 0.01057 |
| 7 | 0-2 | 0.01722 | 0.01612 | 0.01523 | 0.01477 | 0.01433 |
|  | 2-4 | 0.01375 | 0.01133 | 0.01115 | 0.01082 | 0.01050 |
|  | 4-6 | 0.01392 | 0.01247 | 0.01228 | 0.01191 | 0.01155 |
|  | 6-8 | 0.01418 | 0.01297 | 0.01245 | 0.01208 | 0.01172 |
|  | 8-10 | 0.01297 | 0.01326 | 0.01376 | 0.01403 | 0.01431 |
| 8 | 0-2 | 0.01622 | 0.01299 | 0.01319 | 0.01279 | 0.01241 |
|  | 2-4 | 0.02026 | 0.01669 | 0.01644 | 0.01594 | 0.01547 |
|  | 4-6 | 0.02052 | 0.01837 | 0.01809 | 0.01755 | 0.01702 |
|  | 6-8 | 0.02089 | 0.01911 | 0.01835 | 0.01780 | 0.01726 |
|  | 8-10 | 0.01912 | 0.01953 | 0.02027 | 0.02068 | 0.02109 |
